# Switching From Glargine to Degludec: The Effect on Metabolic Control and Safety During 1-Year of Real Clinical Practice in Children and Adolescents With Type 1 Diabetes

**DOI:** 10.3389/fendo.2018.00462

**Published:** 2018-08-23

**Authors:** Barbara Predieri, Tosca Suprani, Giulio Maltoni, Vanna Graziani, Patrizia Bruzzi, Stefano Zucchini, Lorenzo Iughetti

**Affiliations:** ^1^Department of Medical and Surgical Sciences of the Mother, Children and Adults, Pediatric Unit, University of Modena and Reggio Emilia, Modena, Italy; ^2^Department of Pediatrics, Bufalini Hospital, Cesena, Italy; ^3^Department of Pediatrics, S. Orsola-Malpighi Hospital, Bologna, Italy; ^4^Department of Pediatrics, Santa Maria Delle Croci Hospital, Ravenna, Italy

**Keywords:** basal-bolus therapy, type 1 diabetes, insulin degludec, glycemic control, safety, children

## Abstract

**Background/Objective:** Insulin degludec (IDeg) is an ultra-long-acting analog with less daily variability compared to other basal insulins. In this retrospective study we examined 1-year efficacy and safety of IDeg in youth with type 1 diabetes (T1D).

**Subjects/Methods:** Thirty-seven patients [11.7 ± 4.22 years; T1D duration 4.97 ± 3.63 years; once-daily glargine (IGlar) by at least 1 year] were switched to once-daily IDeg because of glycosylated hemoglobin (HbA1c) >7.5% and/or reported physical pain at IGlar injection. Changes in HbA1c, 30-day mean fasting plasma glucose (mean FPG), daily insulin dose, and severe hypoglycemia rates were collected at basal insulin switch (T0), 3-months (T1), 6-months (T2), and 12-months (T3) after IDeg was started.

**Results:** In patients with HbA1c >7.5% at T0 we found a decrease in HbA1c values (%) from 8.46 ± 0.53 to 7.89 ± 0.72 at T1 (*p* = 0.008) and 7.97 ± 0.89 at T2 (*p* = 0.035). At T3, 38.9% of patients had HbA1c ≤ 7.5%. Mean FPG levels significantly decreased at T2 (*p* = 0.043). In the overall study population, we documented an increase in IDeg dose (+12.5% at T3; *p* < 0.001) and a decrease in mealtime insulin dose (−11.6% at T3; *p* = 0.001) after switch. HbA1c levels were unchanged. No episode of severe hypoglycemia was reported.

**Conclusions:** Our data in children and adolescents with T1D suggest that IDeg dose should be increased by 12% and mealtime insulin doses should be lowered by 11% for patients who previously received IGlar. IDeg might be considered useful and well tolerated and it seems to improve the glycemic control compared to IGlar, mainly in patients with poor glycemic control.

## Introduction

Type 1 diabetes (T1D) is the most common endocrine chronic disease in pediatrics and its incidence in Italy shows a linear increasing temporal trend with an annual increment of 2.94% (1). T1D management is yet difficult despite new insulins and advanced technologies.

The Diabetes Control and Complications Trial (2) and its follow-up Epidemiology of Diabetes Interventions and Complications study (3, 4) demonstrated that a good glycemic control can reduce the risk to develop both short- and long-term complications and delay the progression of existing complications in T1D.

In order to optimize the therapeutic management in both children and adolescents, specific guidelines were published by the International Society for Pediatric and Adolescent Diabetes (ISPAD) (5, 6) and the American Diabetes Association (ADA) (7). Basal-bolus intensive treatment with analogs was established as a mainstay of care for all patients with T1D.

Severe hypoglycemia remains a challenge for patients with T1D across their life span and its rates increase with lower glycosylated hemoglobin (HbA1c) levels (8). Parents' and/or patients' fear of severe hypoglycemia is the main barrier to achieve good glycemic control (9) with subsequently increased risk for diabetic ketoacidosis and long-term complications (10).

Advances in basal insulin therapy came with long-acting analogs glargine (IGlar) and detemir. They represented an improvement over intermediate-acting neutral protamine Hagedorn, but there may still be inter- and intra-individual glucose-lowering effect variability from injection to injection which may consequently have an impact on the risk of both hyper- and hypoglycemia (11, 12). Approximately 70-80% of patients using IGlar need a once-daily injection, whereas others require twice-daily injections to cover their 24 h basal insulin supplementation. These latter patients were demonstrated to need a higher dose of insulin to attain optimal glycemic targets and to experience more frequent episodes of hypoglycemia (13).

Degludec (IDeg) is a new, ultra-long-acting form of insulin, developed for once-daily administration. After subcutaneous administration, IDeg forms long chains of multi-hexamers from which monomers are slowly and continuously released and gradually absorbed into the circulation (14). Due to its unique mechanism of protracted and constant absorption rate, the mean terminal half-life of IDeg exceeds 25 h with a duration of action exceeding 42 h allowing, at steady-state, a lower within-patient day-to-day variability (15). The reproducible pharmacodynamic profile of IDeg allowed an improvement of HbA1c (16), a flat and stable glucose-lowering profile (16–20), a lower risk of hypoglycemia (16, 18, 21–25), and greater treatment satisfaction (26) with respect to IGlar. The switch from IGlar to once-daily IDeg also led to significant decrease in insulin total daily dose (TDD) because of reduction in long-acting analog and/or rapid-acting analog doses (16, 19, 23, 27–30).

The ultra-long pharmacokinetic profile of IDeg seen in adults was demonstrated also in children and adolescents with T1D (31–33) and there is great interest in studies testing whether evidence collected from randomized controlled trials translates into the real world. Our preliminary data in children and adolescents with T1D demonstrated that the switch from IGlar to IDeg allowed a significant reduction in insulin TDD due to a reduction in both basal insulin and rapid-acting analog/regular insulin doses at mealtime (MT). Despite the lack of statistical significance, mean HbA1c was decreased by 0.2% point and fasting plasma glucose (FPG) was improved by 9.5%. Body mass index (BMI) z-score did not change and no episode of severe hypoglycemia was reported (34, 35).

We performed this retrospective study wherein children and adolescents with T1D on once-daily IGlar were studied before and after switching to once-daily IDeg, to evaluate its 1-year efficacy and safety.

## Materials and methods

### Patients and study design

This was a 1-year, retrospective, non-randomized, and single-arm study. Subjects were enrolled among children and adolescents with T1D followed at the Pediatric Diabetes Clinic of the University of Modena and Reggio Emilia between November 2015 and July 2016. The switch from once-daily IGlar (Lantus® 100 Units/mL, Sanofi, Paris, France) to once-daily IDeg (Tresiba®100 Units/mL, Novo Nordisk, Bagsvard, Denmark), was suggested by pediatric diabetologist because of HbA1c >7.5% (>58 mmol/mol) and/or self-reported physical pain at IGlar injection. Inclusion criteria were: age 3–18 years, diagnosis of T1D (36) at least 1 year prior to the study, and treatment for at least 1 year with basal-bolus insulin injections with IGlar as the basal insulin and rapid-acting analog and/or regular insulin as the bolus insulin. Exclusion criteria were: other types of diabetes, the presence of other autoimmune diseases and/or chronic complications, concomitant oral antidiabetic drugs, the presence of antibodies to insulin. Moreover, considering that the insulin requirement increases during pubertal development (6), patients with early puberty (Tanner stage 2) and mid puberty (Tanner stage 3) were excluded to avoid influences on results.

Each patient was used as his/her own control and none dropped out during the 1-year study period.

Primary efficacy objectives were the detection of significant changes in (1) glycemic control indexes and (2) insulin TDD and number of insulin injections. Secondary objectives were the evaluation of tolerability and safety, including severe hypoglycemic episodes, adverse events, and auxological data.

This study was carried out in accordance with the recommendations of Ethics Committee of the University of Modena and Reggio Emilia. The protocol was approved by the Ethics Committee of the University of Modena and Reggio Emilia (Protocol Number 149/15). All subjects gave written informed consent in accordance with the Declaration of Helsinki.

### Outcome variables

Information on HbA1c, FPG, insulin TDD, and number of injections, severe hypoglycemia rates, and BMI z-score was collected at baseline in the day of basal insulin switch (IGlarT0 and IDeg T0), 3-months (T1), 6-months (T2), and 12-months (T3) after IDeg was started.

HbA1c was measured at each visit and it was assayed using a validated high-performance liquid chromatography analyzer (Arkray Adams HA-8160; Menarini diagnostics; Florence, Italy). HbA1c target <7.5% (<58 mmol/mol) was used to define an optimal level of control (7, 37).

Mean FPG values were calculated using the last 30 days self-monitoring of blood glucose (SMBG) profiles recorded by glucometer whose data were daily registered on glycemic diary by all patients/parents and downloaded via Diasend®when possible. All patients checked their blood glucose levels at least four times/day: before breakfast, lunch, dinner, and at bedtime as well as when experiencing symptoms of hypoglycemia. No one used a continuous glucose monitoring system during the entire study period.

Data of insulin doses were analyzed considering insulin TDD, basal long-acting analog (IGlar and IDeg), and bolus insulin (rapid-acting analog and/or regular) at MT; they were reported as units (IU) per kg body weight per day. Basal-bolus insulin doses were individually adjusted by the pediatric diabetologist at clinic visit or during a telephone contact, according to SMBG values. In each patient the type of MT insulin preparation was never changed throughout the study period.

The number of daily insulin injections was considered at baseline and at the end of the study.

Severe hypoglycemia was defined as “an event associated with severe neuroglycopenia usually resulting in coma or seizure and requiring parenteral therapy (glucagon or intravenous glucose)” (38). Severe hypoglycemic events were recorded both in the year before and in the year following the switch from IGlar to IDeg.

In addition to the analyses on the overall population, patients were divided into two groups according to baseline HbA1c values (Group A ≤7.5%; Group B >7.5%) for further comparisons.

### Statistical analyses

Descriptive data are reported as mean ± standard deviation (SD), median, and range. Data were checked for normal distribution using the Kolmogorov-Smirnov test. Longitudinal changes were analyzed using the Friedman's ANOVA for multiple dependent samples, the Wilcoxon Matched Pairs Test for two dependent samples, and McNemar's test. Between-group comparisons were performed using the Mann-Whitney's *U*-test and the Pearson χ^2^. Potential predictors of longitudinal changes in HbA1c were evaluated using a multivariate regression model including gender, age, duration of diabetes, baseline HbA1c, basal, and bolus insulin dose before switching, percentage change in basal insulin dose at the time of switching, and number of daily insulin injections before switching. For each test, statistical significance was considered for *p* < 0.05. Statistical analyses were performed using the STATISTICA™ software (StatSoft Inc., Tulsa, OK, USA).

## Results

### Patient characteristics

This study presents data from 37 children and adolescents (22 males, 59.5%) with T1D, having age of 11.7 ± 4.22 years (median 12.7 years; range 3.1–17.9 years) and duration of diabetes of 4.97 ± 3.63 years (median 4.3 years; range 1.0–14.4 years). Eighteen patients (48.6%) were recruited because of HbA1c >7.5% (>58 mmol/mol), while 19 patients self-reported physical pain to IGlar injection despite a good glycemic control. At the beginning of the study, 51.3% of subjects were in a prepubertal status (Tanner stage 1) and none started puberty during the 1-year follow-up. All the other 18 patients were post-pubertal (Tanner stage 4–5). Nine out 18 were females and all had menarche.

All patients received IDeg injection during the evening or before bedtime and 8 patients received regular insulin injections at breakfast and lunch while they used rapid-acting analog at dinner. All other patients used a rapid-acting analog at MT. The initial dose of IDeg ranged from 60 to 150% of the dose of IGlar in 26 patients, while the initial dose of the bolus insulin was not changed in 29. Baseline characteristics of study subjects are reported in Table 1.

**Table 1:** Characteristics of study population.

**Characteristics**	**Basal insulin switch**		**IDeg follow-up**			**χ^2^**	**p**
	**IGlar T0**	**IDeg T0**	**T1**	**T2**	**T3**		
Puberty (Tanner 1/Tanner 4-5)	19/18	=	=	=	=	–	–
HbA1c (%)	7.56 ± 1.04 (7.50)	–	7.35 ± 0.97 (7.20)	7.43 ± 1.02 (7.30)	7.49 ± 1.15 (7.30)	1.50	0.682
HbA1c (mmol/mol)	59.1 ± 11.3 (58.5)		56.8 ± 10.6 (55.2)	57.7 ± 11.1 (56.3)	58.3 ± 12.5 (56.3)		
HbA1c ≤7.5% (≤58mmol/mol)		-					
T0 vs. T1	19 (51.4%)	-	20 (54.1%)			0.00	1.000
T0 vs. T2	19 (51.4%)	-		22 (59.5%)		0.31	0.579
T0 vs. T3	19 (51.4%)				23 (62.2%)	0.90	0.343
Mean FPG (mg/dl)	168.5 ± 46.6 (163.0)	–	161.8 ± 42.8 (152.5)	141.6 ± 21.3 (138.5)	143.8 ± 18.6 (143.0)	2.90	0.400
TDD insulin (IU/kg/day)	0.93 ± 0.26 (0.95)	0.90 ± 0.27 (0.94)	0.89 ± 0.25 (0.88)	0.89 ± 0.26 (0.90)	0.91 ± 0.23 (0.93)	8.97	0.062
Basal insulin (IU/kg/day)	0.38 ± 0.12 (0.36)	0.35 ± 0.12 (0.34)	0.39 ± 0.11 (0.36)	0.40 ± 0.11 (0.41)	0.42 ± 0.11 (0.42)	20.6	<**0.001**
MT insulin (IU/kg/day)	0.55 ± 0.21 (0.52)	0.55 ± 0.20 (0.52)	0.50 ± 0.19 (0.49)	0.49 ± 0.21 (0.50)	0.49 ± 0.19 (0.45)	17.5	**0.001**
Insulin injections (n/day)	5.23 ± 1.18 (5.50)	–	–	–	4.89 ± 1.02 (4.00)	–	**0.002**
Severe hypoglycemic events (*n*)	1/37	–	0/37	0/37	0/37	–	–
BMI z-score (SDS)	0.41 ± 1.00 (0.33)	–	0.39 ± 1.00 (0.49)	0.40 ± 0.98 (0.43)	0.40 ± 1.06 (0.65)	1.19	0.755

*Bold values indicate statistically significant p < 0.05*.

### Glycemic control

**Figure 1:**
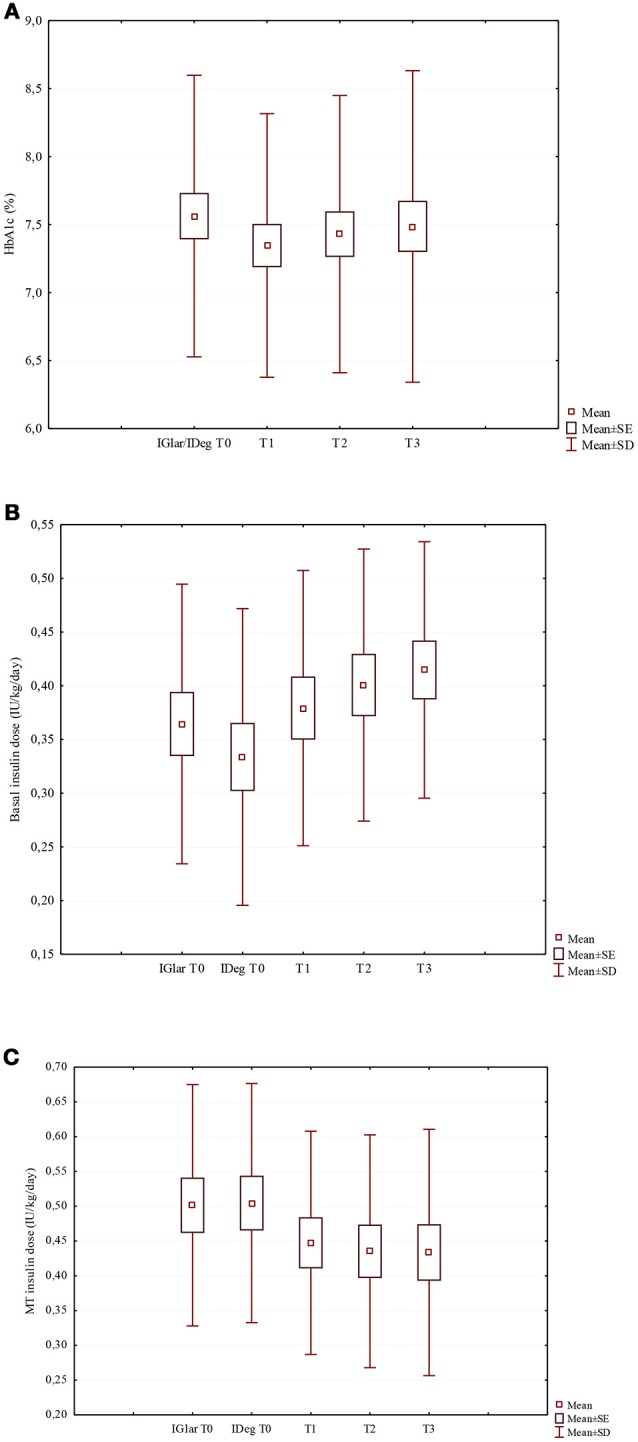
Changes of HbA1c **(A)**, basal insulin dose **(B)**, and MT insulin dose **(C)** in the study population after the switch from IGlar to IDeg. **(A)** HbA1c (%) (χ^2^ = 1.50; *p* = 0.682). **(B)** Basal insulin dose (IU/kg/day) (χ^2^ = 20.6; *p* < 0.001). **(C)** MT insulin dose (IU/kg/day) (χ^2^ = 17.5; *p* = 0.001).

After switching to IDeg, despite the lack of statistical significance, HbA1c median values decreased from 7.5% (58 mmol/mol) to 7.2% (55 mmol/mol) at T1 and 7.3% (56 mmol/mol) at both T2 and T3 (Table 1; Figure 1A). At T0, 19 out of 37 patients (51.4%) had a good HbA1c level, while at T3 HbA1c values were on target in 62.2% of the patients (Figure S1).

Mean FPG levels were not significantly decreased, but at T3 values resulted 3% lower compared to T0 (Table 1).

Glycemic control indexes at the beginning and at the end of the study period were not different between subjects according to pubertal status. Moreover, no significant longitudinal change was demonstrated in both prepubertal and post-pubertal groups (data not shown). Median HbA1c values at the end of the study were 6.8% (51 mmol/mol) in prepubertal subjects and 7.4% (57 mmol/mol) in post-pubertal ones.

### Insulin requirement

The switch from IGlar to IDeg allowed a significant decrease of the number of daily insulin injections from T0 to T3 (5.23 ± 1.18 vs. 4.89 ± 1.02, respectively; *p* = 0.002).

The insulin TDD was slightly lower during the IDeg administration period than in the IGlar one, although the difference did not reach statistical significance (*p* = 0.062). Looking at IDeg and MT apart, a significant increase in the IDeg dose (*p* < 0.001) and a significant decrease in MT insulin dose (*p* = 0.001) (Table 1; Figures 1B,C, respectively) were documented. At T3 the median IDeg dose increased by 12.5%, while the median MT insulin dose decreased by 11.6%. We found that the distribution ratio of basal insulin dose with respect to the insulin TDD significantly increased from 41.4 ± 9.7% with IGlar at T0 to 46.9 ± 11.8% with IDeg at T3 (*p* = 0.004).

When analyzing data according to pubertal status, we found that TDD was always significantly higher in post-pubertal group respect to prepubertal one, as expected (data not shown). Using Friedman ANOVA we demonstrated a significant decrease of TDD only in post-pubertal patients (χ^2^ = 10.8; *p* = 0.029) and this change was due to the significant reduction of MT insulin dose (χ^2^ = 14.1; *p* = 0.007). In prepubertal subjects TDD was longitudinally unchanged, but the basal:bolus ratio was changed because of IDeg dose significantly increased (χ^2^ = 15.3; *p* = 0.004).

### Safety data

One severe hypoglycemic event was reported before basal insulin switching, while none occurred in the 1-year follow-up with IDeg treatment.

No side effect was reported during the study period and patients who had previously complained physical pain at IGlar injection did not present this painful symptom using IDeg.

Finally, the BMI z-score remained unchanged after switching to IDeg (Table 1).

### Multiple regression analysis

Considering the whole study population, multivariate regression analysis allowed us to identify HbA1c value at T0 as a predictor of HbA1c change at T1 (β = −0.667, *p* = 0.001) and T2 (β = −0.681, *p* = 0.002); none of the variables tested predicted HbA1c change at T3 (Table S1).

### Subgroup analysis according to HBA1c group

#### Patient characteristics

The statistical analysis was also performed subdividing the study population into 2 groups:

- HbA1c ≤7.5% (≤58 mmol/mol) including 19 patients (12 males, 63.1%) aged 10.2 ± 4.04 years (median 8.70 years; range 3.60-17.1 years) and duration of diabetes of 4.56 ± 3.98 years (median 2.70 years; range 1.10–14.4 years); 12 (63.1%) in prepubertal status.
- HbA1c >7.5% (>58 mmol/mol) including 18 patients (10 males, 55.5%) aged 13.3 ± 3.87 years (median 13.9 years; range 3.1–17.9 years) and duration of diabetes of 5.41 ± 3.27 years (median 5.25 years; range 1.0–12.0 years); 7 (38.9%) in prepubertal status.

Baseline characteristics of each group are reported in Table 2. Despite patients included in Group A were significantly younger than those in Group B (*p* = 0.013) the duration of diabetes was not different (*p* = 0.267).

**Table 2:** Characteristics of patients according to HbA1c group.

**Characteristics**	**Study time**	**Group**		***p***
		**A. HbA1c ≤ 7.5% (≤ 58 mmol/mol)**	**B. HbA1c >7.5% (> 58 mmol/mol)**	
Subjects (*n*)	IGlar/IDeg T0	19	18	–
Gender (Male/Female)	IGlar/IDeg T0	12/7	10/8	0.638
Age (years)	IGlar/IDeg T0	10.2 ± 4.04 (8.70)	13.3 ± 3.87 (13.9)	**0.013**
Duration of diabetes (years)	IGlar/IDeg T0	4.56 ± 3.98 (2.70)	5.41 ± 3.27 (5.25)	0.267
Puberty (No/Yes)	IGlar/IDeg T0	12/7	7/11	0.139
HbA1c (%) (mmol/mol)	IGlar/IDeg T0 T1 T2 T3	6.71 ± 0.55 (6.80) 49.8 ± 6.0 (50.8) 6.83 ± 0.90 (6.70) 51.2 ± 9.8 (49.7) 6.92 ± 0.87 (6.70) 52.1 ± 9.5 (49.7) 6.88 ± 0.78 (6.60) 51.7 ± 8.6 (48.6) χ^2^ = 5.41; *p =* 0.144	8.46 ± 0.53 (8.50) 68.9 ± 5.8 (69.4) 7.89 ± 0.72 (7.85) 62.7 ± 7.9 (62.3) 7.97 ± 0.89 (7.80) 63.6 ± 9.8 (61.7) 8.12 ± 1.13 (7.70) 65.3 ± 12.4 (60.6) χ^2^ = 5.28; *p =* 0.152	<**0.001** **0.001** **0.002** **0.001**
Mean FPG (mg/dl)	IGlar/IDeg T0 T1 T2 T3	145.3 ± 22.1 (141.5) 141.5 ± 17.5 (137.0) 136.7 ± 16.3 (132.5) 138.6 ± 17.7 (137.0) χ^2^ = 0.64; *p =* 0.888	204.5 ± 52.7 (195.0) 191.1 ± 52.1 (186.0) 153.2 ± 28.6 (152.5) 160.5 ± 10.3 (158.0) χ^2^ = 4.66; *p =* 0.199	**0.001** **0.011** 0.201 **0.042**
TDD insulin (IU/kg/day)	IGlar T0 IDeg T0 T1 T2 T3	0.86 ± 0.25 (0.85) 0.84 ± 0.25 (0.83) 0.83 ± 0.23 (0.78) 0.83 ± 0.21 (0.85) 0.85 ± 0.21 (0.83) χ^2^ = 4.49; *p =* 0.343	1.00 ± 0.26 (1.00) 0.97 ± 0.28 (0.99) 0.95 ± 0.27 (0.92) 0.95 ± 0.29 (0.95) 0.98 ± 0.23 (0.98) χ^2^ = 5.46; *p =* 0.243	0.186 0.098 0.242 0.230 0.118
Basal insulin (IU/kg/day)	IGlar T0 IDeg T0 T1 T2 T3	0.36 ± 0.13 (0.35) 0.33 ± 0.14 (0.31) 0.38 ± 0.13 (0.35) 0.40 ± 0.13 (0.38) 0.41 ± 0.12 (0.42) χ^2^=**11.1;** ***p** =* **0.026**	0.39 ± 0.11 (0.40) 0.37 ± 0.10 (0.40) 0.40 ± 0.09 (0.40) 0.40 ± 0.10 (0.42) 0.42 ± 0.09 (0.43) χ^2^=**9.93;** ***p** =* **0.042**	0.403 0.176 0.370 0.750 0.843
MT insulin (IU/kg/day)	IGlar T0 IDeg T0 T1 T2 T3	0.50 ± 0.17 (0.47) 0.50 ± 0.17 (0.47) 0.45 ± 0.16 (0.42) 0.43 ± 0.17 (0.42) 0.43 ± 0.18 (0.40) χ^2^=**11.7;** ***p** =* **0.020**	0.61 ± 0.23 (0.58) 0.60 ± 0.22 (0.60) 0.55 ± 0.20 (0.52) 0.55 ± 0.23 (0.54) 0.56 ± 0.19 (0.57) χ^2^ = 7.11; *p =* 0.130	0.062 0.104 0.095 0.158 0.058
Insulin injections (n/day)	IGlar/IDeg T0 T3	5.66 ± 1.20 (6.00) 5.26 ± 1.04 (6.00) ***p** =* **0.012**	4.78 ± 0.99 (4.00) 4.50 ± 0.86 (4.00) *p =* 0.068	**0.023** **0.043**
Severe hypoglycemic events (n)	IGlar/IDeg T0 T1 T2 T3	1/19 0/19 0/19 0/19	0/18 0/18 0/18 0/18	– – – –
BMI z-score (SDS)	IGlar/IDeg T0 T1 T2 T3	0.39 ± 0.99 (0.32) 0.34 ± 1.06 (0.49) 0.41 ± 1.05 (0.60) 0.41 ± 1.11 (0.80) χ^2^ = 7.47; *p =* 0.058	0.43 ± 1.05 (0.39) 0.44 ± 0.96 (0.43) 0.38 ± 0.94 (0.40) 0.40 ± 1.04 (0.63) χ^2^ = 2.47; *p =* 0.481	0.939 0.976 0.750 0.704

*Bold values indicate statistically significant p < 0.05*.

#### Glycemic control

As expected, Group A showed significantly lower levels of HbA1c compared to Group B at T0 and throughout the study period (Table 2). After the switch from IGlar to IDeg, 11 patients of Group B (61.1%) improved their HbA1c values and 7 (38.9%) reached HbA1c levels ≤7.5% (≤58 mmol/mol) at T3. In Group A, 16 patients (84.2%) kept HbA1c ≤7.5% at T3 (Figure S1). As compared to T0, HbA1c was unchanged after switching to IDeg in both groups. In Group B, we found a decrease in HbA1c values from 8.46 ± 0.53% (68.9 ± 5.8 mmol/mol) to 7.89 ± ± 0.72% (62.7 ± 7.9 mmol/mol) at T1 (*p* = 0.008), 7.97 ± 0.89% at T2 (63.6 ± 9.8 mmol/mol) (*p* = 0.035), and 8.12 ± 1.13% (65.3 ± 12.4 mmol/mol) at T3 (*p* = 0.136; Figure S2).

Mean FPG values were also significantly lower in Group A compared to Group B (Table 2). In Group B, mean FPG levels significantly decreased from 204.5 ± 52.5 mg/dl at T0 to 153.2 ± 28.6 mg/dl at T2 (*p* = 0.043; Figure S2), with percent decreases of mean FPG values of −10.4% at T1, −18.2% at T2, and −8.26% at T3.

#### Insulin requirement

Throughout the study period, Group A patients did more daily insulin injections than those in Group B. The switch from IGlar to IDeg allowed a statistical significant decreased in the number of injections at the end of follow-up only in Group A (*p* = 0.012; Table 2).

Insulin doses were never significantly different between analyzed groups. After switching to IDeg, insulin TDD remained unchanged in both groups. Looking at basal insulin and MT apart, we documented a significant increase of IDeg dose in both Group A and Group B (*p* = 0.026 and *p* = 0.042, respectively), while a significant decrease of MT insulin dose was found only in Group A (*p* = 0.020; Table 2). Specifically, at T3, the IDeg dose increased by 13.4% in Group A and 8.3% in Group B. The MT insulin dose was decreased by 12.7% in Group A and 7.9% in Group B. The distribution ratio of basal insulin respect to insulin TDD was not different between Group A and Group B both at T0 (IGlar T0: 42.4 ± 8.54 vs. 40.2 ± 10.8%, respectively; *p* = 0.438) and at T3 (IDeg T3: 49.9 ± 13.4 vs. 43.8 ± 9.36%, respectively; *p* = 0.438).

#### Safety data

The only severe hypoglycemic event during IGlar treatment was reported by a patient belonging to Group A. No severe hypoglycemic event occurred in the 1-year follow-up with IDeg in both groups.

After switching to IDeg, the BMI z-score remained unchanged in both groups (Table 2).

## Discussion

In this study, children and adolescents outpatients with T1D whose once-daily IGlar was switched to once-daily IDeg were observed for 1-year. Our data provide a better understanding on how to change both basal and bolus insulin doses when switching from IGlar to IDeg and how these therapeutic changes can impact on glycemic control, in a real-world pediatric clinical practice.

Despite IDeg pharmacokinetic profile seen in adults was shown also in children and adolescents with T1D (31), to the best of our knowledge only one published study compared its efficacy and safety with respect to IGlar in this age group (33).

Appropriate basal and bolus insulin dosages need be adjusted when switching to IDeg; we found a significant increase in IDeg dose (+12.5%) compared to IGlar dose and a significant reduction in MT insulin doses (−11.6%). The increase in IDeg dose is at variance with a published study on adults with T1D, where mean doses of basal and pre-meal bolus insulin were significantly decreased by 14% and 10% in the IDeg group compared with the IGlar group (22). It was suggested that appropriate replacement IDeg doses should be lowered by 10–20% for patients who previously received once-daily injection of IGlar and by 20–30% for those previously given twice-daily injections (39). Based on our data, more studies are needed to verify what is the best way to switch from IGlar to IDeg in pediatric population.

Despite precise recommendations cannot be made on the basis of our data, we demonstrated that the mean final ratio of IDeg was 50% of the insulin TDD, suggesting that this probably should be the optimal starting IDeg dose in a basal-bolus treatment to obtain a good glycemic control. According to ISPAD recommendations, in multiple daily insulin injections therapy, 40–60% of the insulin TDD should be given as a long-acting analog (6).

However, our results should be also interpreted considering that both early and mid pubertal patients were not included in the population study avoiding data associated with the worst insulin resistance and the necessary increase of TDD (6). Basal insulin dose for T1D treated with multiple daily injections should be modified according to patient age and pubertal status. Subjects having an age between 10 and <20 years were demonstrated to have a significantly higher percentage of basal insulin dose than both younger and older ones (40). We found that basal insulin dose was not different between pre-pubertal and post-pubertal patients, but during 1-year follow-up it was significantly increased in prepubertal group but not in the post-pubertal ones. Considering also the metabolic control obtained by our post-pubertal patients, we hypothesize that we should consider the possibility to increase more the IDeg dose even in these patients, without taking into consideration data provided by clinical trials in adults.

In our study population, HbA1c and mean FPG concentrations were unchanged after switching to IDeg. In children with HbA1c ≤7.5% at baseline, the 1-year switching to IDeg had no effect on both HbA1c (+0.10%) and mean FPG values (−2.7%), making questionable the usefulness of changing an insulin therapy that has already allowed a good glycemic control. On the other hand, these patients were switched to IDeg because of self-reported physical pain at IGlar injection, which was not referred during follow-up. This aspect is probably important for a better quality of life. Benefits were instead noted in the group of patients with high basal HbA1c levels, both in terms of HbA1c and mean FPG. At 1-year follow-up, in these patients HbA1c decreased by 0.55% point with IDeg, in agreement with data in adults with T1D, and mean FPG levels decreased by 18.2% after 6 months and 8.3% at 1-year follow-up. Reaching the HbA1c goal is important to avoid the long-term microvascular and macrovascular complications of T1D while also avoiding acute complication such as hypoglycemia. Moreover, at the end of our study, 62.2% of all patients had HbA1c values at target, as compared to 43% in adults (22). This is the first report showing in children and adolescents with T1D that baseline HbA1c was a significant predictor of change in HbA1c when switching from IGlar to IDeg. Larger decreases in HbA1c with higher baseline HbA1c were previously demonstrated in adults (41). To attain these results on glycemic control insulin doses were personalized as possible and adjusted according to individual needs. However, some of the poorly controlled patients experienced no improvement in HbA1c levels after IDeg, suggesting that switching to a new insulin by itself does not necessarily improve glucose control.

The switch from IGlar to IDeg in our patients allowed a significant reduction of the number of daily insulin injections at the end of follow-up. These findings, already reported in adults with T1D (29), are important considering that insulin effectiveness can be reduced by several factors related to patients' practices in taking their daily dose.

No severe hypoglycemic episode was documented in our study after switching from IGlar to IDeg. Nevertheless, despite we can exclude problems with underreporting for the severe hypoglycemia, milder symptomatic hypoglycemia was not assessed. In children it was demonstrated that no severe hypoglycemia occurred with both IGlar and IDeg, but nocturnal hypoglycemia significantly decreased only with IDeg use (33).

Strengths of our study include the long follow-up time and the use of real-life data, which are more representative of the outpatient population than those included in clinical trials. However, this study has limitations such as the retrospective, non-randomized nature of the design study. First of all, a control group was not included. However, we believe that our patients with their baseline features, recorded before the switch from IGlar to IDeg, could be considered as control of themselves. Our main outcome was to evaluate the efficacy and safety of IDeg in a population that was previously in therapy with IGlar, comparing metabolic and therapeutic data before and after the switch. Being patients the same before and after the basal insulin switch, probably we avoided the bias of different individual ability to manage therapeutic and metabolic control due to different understanding of therapeutic education and adaptation to T1D. Secondly, we recruited only pre- and post-pubertal subjects. We should take into consideration that insulin daily requirement is different between pre-puberty and different stages of pubertal development, during which TDD gradually rise. The correct dose of insulin is the one which achieves the best attainable glycemic control for an individual child or adolescent without causing obvious hypoglycemia problems. So, insulin doses must be always personalized as possible and adjusted according to individual needs, including pubertal status. Third aspect to be considered is that the decision to switch to IDeg based on HbA1c levels ≤7.5% (≤ 58 mmol/mol) accounts for the 51.4% of our study population, with a possible bias toward selecting patients with a good compliance having already a good glycemic control. Moreover, insulin titration was performed according to routine clinical practice without reference to a pre-specified algorithm, thus potentially increasing heterogeneity. Finally, daily blood glucose levels were measured using portable glucometers. Although patients and parents were well trained and had already used these glucose meters for some years before enrollment, possible errors and failures in measuring and recording blood glucose levels could have occurred. Despite these limitations, our results are peculiar and innovative with respect to adult experience and the follow-up is sufficiently long to avoid influences by the period of titration of IDeg.

In conclusion, in our real-world clinical practice switching to IDeg resulted effective and safe in children and adolescents with T1D. On the basis of our experience the IDeg dose should be increased by 12.5% and MT replacement doses should be reduced by 11.6% in patients who previously received IGlar. The basal:bolus ratio 50:50 seems to be appropriate to obtain a reduction in the number of insulin injections and an improvement of glycemic control without increasing the risk for severe hypoglycemic events. IDeg may represent an ultra-long-acting analog alternative to IGlar for patients with a poor glycemic control and/or with physical pain at IGlar injection.

## Author contributions

BP acts as guarantor for the contents of this article. All authors (BP, TS, GM, VG, PB, SZ, and LI) contributed substantially to study design, data collection, statistical analysis and interpretation of the data, manuscript writing and approved the final version to be published.

### Conflict of interest statement

The authors declare that the research was conducted in the absence of any commercial or financial relationships that could be construed as a potential conflict of interest.

## Abbreviations

ADA: American Diabetes Association
BMI: Body mass index
FPG: Fasting plasma glucose
HbA1c: Glycosylated hemoglobin
IDeg: Insulin degludec
IGlar: Insulin glargine
ISPAD: International Society for Pediatric and Adolescent Diabetes
IU: International unit
MT: Mealtime
n: Number
SD: Standard deviation
SDS: Standard deviation score
SE: Standard error
SMBG: Self-monitoring of blood glucose
T0: Switch basal insulin time
T1: 3 months after insulin switch
T2: 6 months after insulin switch
T3: 12 months after insulin switch
T1D: Type 1 diabetes
TDD: Total daily dose
–: Not determined
=: The same.
