# Supplementary material for: Switching From Glargine to Degludec: The Effect on Metabolic Control and Safety During 1-Year of Real Clinical Practice in Children and Adolescents With Type 1 Diabetes
Source: Front Endocrinol (Lausanne). 2018 Aug 23;9:462. doi: 10.3389/fendo.2018.00462 (PMC6115523; doi:10.3389/fendo.2018.00462)

SUPPLEMENTARY MATERIAL

TABLES

Table S1 - Multiple regression analysis: predictive factors for % point changes in HbA1c.

HbA1c (%)	Multiple R	Multiple R^2	p
**T1 - T0**	**0.661**	**0.437**	**0.024**
**T2 - T0**	0.598	0.358	0.091
**T3 - T0**	0.449	0.202	0.541

HbA1c (%) T1 - T0	Param.	SE	p	±95CI	β	β SE	±95CI
**Intercept**	**3.816**	**1.461**	**0.014**	**[0.823][6.810]**			
**Gender (Male)**	-0.051	0.112	0.649	[-0.280][0.177]	-0.068	0.148	[-0.371][0.235]
**Age (years)**	**0.085**	**0.036**	**0.025**	**[0.011][0.158]**	**0.478**	**0.202**	**[0.065][0.892]**
**Duration of diabetes (years)**	**-0.081**	**0.039**	**0.049**	**[-0.162][-0.000]**	**-0.392**	**0.191**	**[-0.784][-0.000]**
**HbA1c at T0 (%)**	**-0.485**	**0.138**	**0.001**	**[-0.769][-0.201]**	**-0.667**	**0.190**	**[-1.058][-0.277]**
**IGlar T0 (IU/kg/day)**	0.710	1.087	0.519	[-1.516][2.936]	0.112	0.172	[-0.240][0.466]
**MT insulin at IGlar T0 (IU/kg/day)**	-0.462	0.553	0.410	[-1.595][0.671]	-0.127	0.152	[-0.439][0.185]
**IDeg T0 - IGlar T0 (%)**	-0.005	0.006	0.395	[-0.018][0.007]	-0.128	0.149	[-0.433][0.176]
**Daily insulin injections (n)**	-0.189	0.129	0.157	[-0.455][0.077]	-0.295	0.203	[-0.712][0-120]
**HbA1c (% point) T2 - T0**	**Param.**	**SE**	**P**	**±95CI**	**β**	**β SE**	**±95CI**
**Intercept**	**4.786**	**1.609**	**0.006**	**[1.489][8.083]**			
**Gender (Male)**	-0.190	0.123	0.135	[-0.442][0.062]	-0.243	0.158	[-0.567][0.080]
**Age (years)**	0.046	0.039	0.250	[-0.035][0.127]	0.253	0.215	[-0.188][0.694]
**Duration of diabetes (years)**	-0.018	0.043	0.688	[-0.107][0.071]	-0.083	0.204	[-0.501][0.336]
**HbA1c at T0 (%)**	**-0.510**	**0.152**	**0.002**	**[-0.823][-0.198]**	**-0.681**	**0.203**	**[-1.097][-0.264]**
**IGlar T0 (IU/kg/day)**	-0.448	1.197	0.711	[-2.900][2.004]	-0.069	0.184	[-0.446][0.308]
**MT insulin at IGlar T0 (IU/kg/day)**	-0.106	0.609	0.863	[-1.354][1.142]	-0.028	0.163	[-0.361][0.305]
**IDeg T0 - IGlar T0 (%)**	-0.006	0.007	0.339	[-0.021][0.007]	-0.154	0.159	[-0.480][0.171]
**Daily insulin injections (n)**	-0.246	0.143	0.096	[-0.539][0.046]	-0.373	0.217	[-0.818][0.071]
**HbA1c (% point) T3 - T0**	**Param.**	**SE**	**P**	**±95CI**	**β**	**β SE**	**±95CI**
**Intercept**	2.143	1.717	0.222	[-1.374][5.661]			
**Gender (Male)**	-0.212	0.131	0.118	[-0.481][0.057]	-0.284	0.176	[-0.645][0.077]
**Age (years)**	-0.006	0.042	0.894	[-0.092][0.081]	-0.032	0.240	[-0.524][0.459]
**Duration of diabetes (years)**	0.060	0.046	0.208	[-0.035][0.155]	0.294	0.228	[-0.173][0.761]
**HbA1c at T0 (%)**	-0.222	0.162	0.183	[-0.555][0.111]	-0.309	0.227	[-0.774][0.155]
**IGlar T0 (IU/kg/day)**	-0.498	1.277	0.699	[-3.115][2.118]	-0.080	0.205	[-0.501][0.340]

**MT insulin at IGlar T0 (IU/kg/day)**	-0.439	0.650	0.504	[-1.771][0.892]	-0.122	0.181	[-0.494][0.249]
**IDeg T0 - IGlar T0 (%)**	-0.003	0.007	0.675	[-0.019][0.012]	-0.075	0.177	[-0.438][0.288]
**Daily insulin injections (n)**	-0.061	0.152	0.692	[-0.374][0.252]	-0.097	0.242	[-0.592][0.399]

FIGURE LEGENDS

Figure S1 - HbA1c changes in study population 1-year after the switch from IGlar to IDeg. Each marker represents an individual patient.

Figure S2 - Glycemic control indexes changes (HbA1c - Panel A; FPG - Panel B) after the switch from IGlar to IDeg in both Group A and Group B (mean; ±SE; ±2SD)

FIGURES

Figure S1

A - HbA1c % value

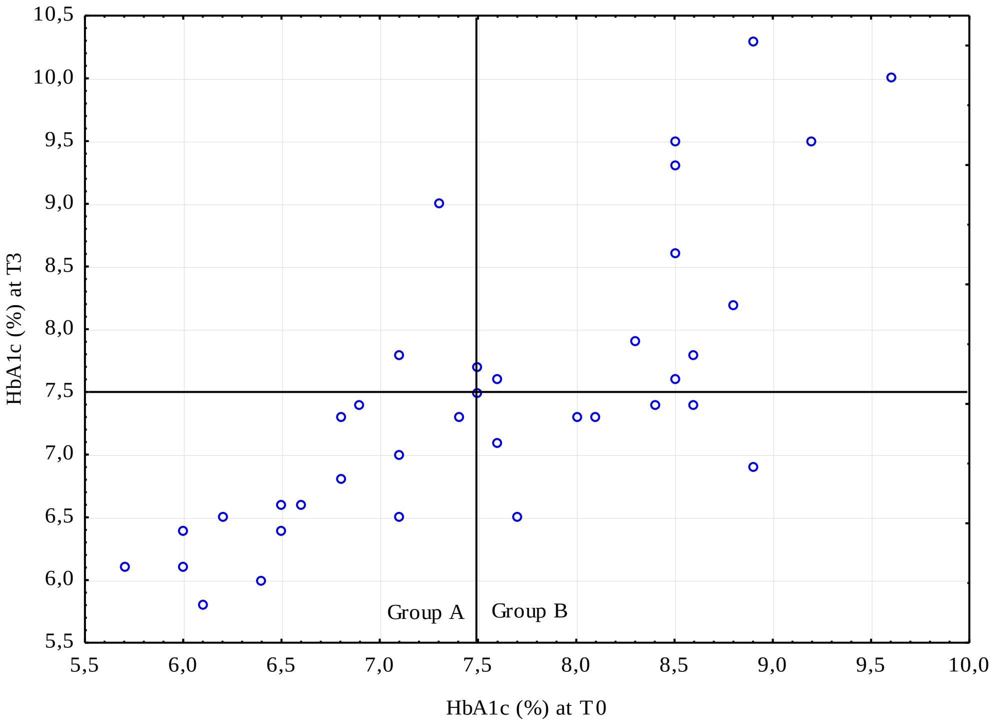

B - HbA1c % changes

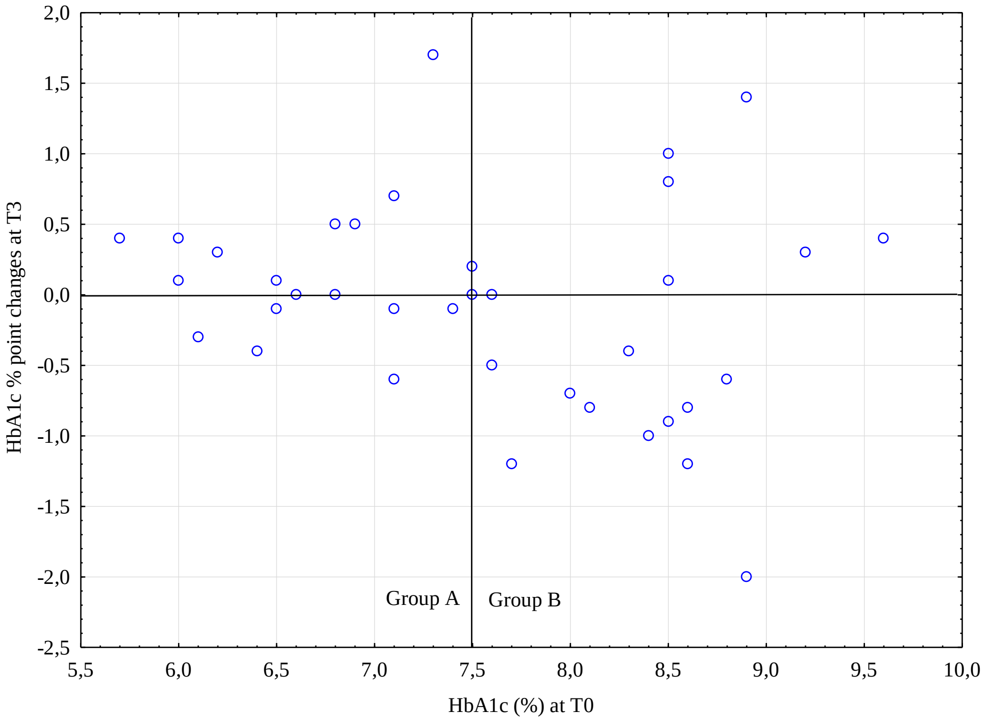

Figure S2

A - HbA1c (%)

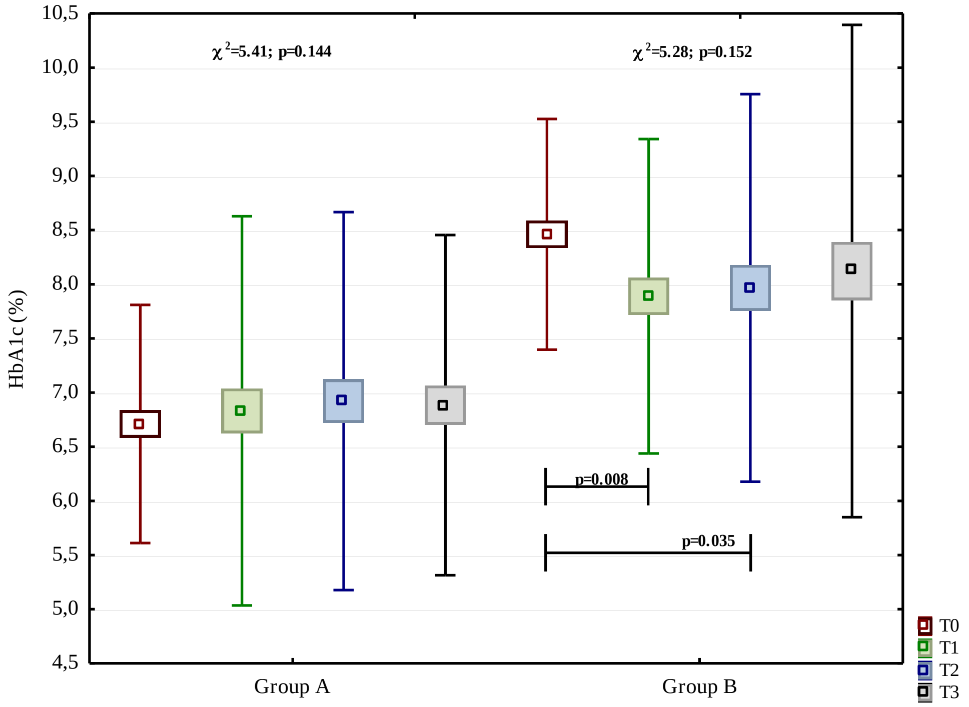

B - Mean FPG (mg/dl)

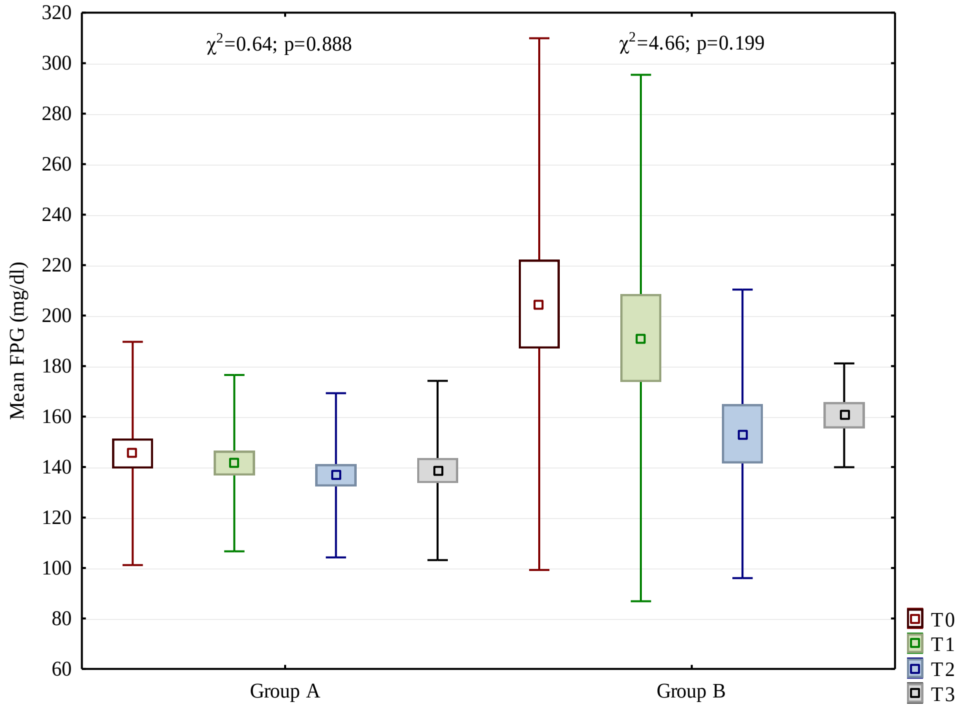

Supplement: Supplementary file 1 [file Data_Sheet_1.PDF]
